# Hypophosphatemia is an independent risk factor for AKI among hospitalized patients with COVID-19 infection

**DOI:** 10.1080/0886022X.2021.1979039

**Published:** 2021-09-19

**Authors:** Zijin Chen, Chenni Gao, Haijin Yu, Lin Lu, Jialin Liu, Wei Chen, Xiaogang Xiang, Hafiz Muhammad Jafar Hussain, Benjamin J. Lee, Chuanlei Li, Wenjie Wei, Yuhan Huang, Xiang Li, Zhengying Fang, Shuwen Yu, Qinjie Weng, Yan Ouyang, Xiaofan Hu, Jun Tong, Jian Liu, Li Lin, Mingyu Liu, Xiaoman Xu, Dan Liu, Yuan Song, Xifeng Lv, Yixin Zha, Zhiyin Ye, Tingting Jiang, Jieshuang Jia, Xiaonong Chen, Yufang Bi, Jun Xue, Nan Chen, Weiguo Hu, Cijiang John He, Huiming Wang, Jun Liu, Jingyuan Xie

**Affiliations:** a Department of Nephrology, Institute of Nephrology, Ruijin Hospital, Shanghai Jiao Tong University, School of Medicine, Shanghai, China; b Department of Nephrology, North Huashan Hospital, Fudan University, Shanghai, China; c Department of Critical Care Medicine, Ruijin Hospital, Shanghai Jiao Tong University, School of medicine, Shanghai, China; d Department of Pulmonary and Critical Care Medicine, Ruijin Hospital, Institute of Respiratory Diseases, Shanghai Jiao Tong University, School of Medicine, Shanghai, China; e Department of Infectious Diseases, Ruijin Hospital, Shanghai Jiao Tong University, School of Medicine, Shanghai, China; f Houston Kidney Consultants, Houston, TX, USA; g Houston Methodist Institute for Academic Medicine, Houston, TX, USA; h Department of Nephrology, Shanghai General Hospital, Shanghai Jiao Tong University, Shanghai, China; i Wuhan Ninth Hospital, Wuhan, China; j Renal Department, Wuhan Ninth Hospital, Wuhan, Hubei, China; k Radiology Department of Renmin Hospital, Wuhan University, Wuhan, Hubei, China; l Renal Department of Renmin Hospital, Renmin Hospital of Wuhan University, Wuhan, Hubei, China; m Clinical Research Center, Shanghai General Hospital, Shanghai Jiao Tong University, Shanghai, China; n Department of Endocrinology and Metabolism disease, Ruijin Hospital, Shanghai Jiao Tong University, School of Medicine, Shanghai, China; o Department of Nephrology, Huashan Hospital, Fudan University, Shanghai, China; p Department of Surgery, Ruijin Hospital, Shanghai Jiao Tong University School of Medicine, Shanghai, China; q Division of Nephrology, Mount Sinai School of Medicine, New York, NY, USA

**Keywords:** COVID-19, proximal tubule, acute kidney injury, risk factors, hypophosphate

## Abstract

**Background:**

This study sought to investigate incidence and risk factors for acute kidney injury (AKI) in hospitalized COVID-19.

**Methods:**

In this retrospective study, we enrolled 823 COVID-19 patients with at least two evaluations of renal function during hospitalization from four hospitals in Wuhan, China between February 2020 and April 2020. Clinical and laboratory parameters at the time of admission and follow-up data were recorded. Systemic renal tubular dysfunction was evaluated *via* 24-h urine collections in a subgroup of 55 patients.

**Results:**

In total, 823 patients were enrolled (50.5% male) with a mean age of 60.9 ± 14.9 years. AKI occurred in 38 (40.9%) ICU cases but only 6 (0.8%) non-ICU cases. Using forward stepwise Cox regression analysis, we found eight independent risk factors for AKI including decreased platelet level, lower albumin level, lower phosphorus level, higher level of lactate dehydrogenase (LDH), procalcitonin, C-reactive protein (CRP), urea, and prothrombin time (PT) on admission. For every 0.1 mmol/L decreases in serum phosphorus level, patients had a 1.34-fold (95% CI 1.14–1.58) increased risk of AKI. Patients with hypophosphatemia were likely to be older and with lower lymphocyte count, lower serum albumin level, lower uric acid, higher LDH, and higher CRP. Furthermore, serum phosphorus level was positively correlated with phosphate tubular maximum per volume of filtrate (TmP/GFR) (Pearson *r* = 0.66, *p* < .001) in subgroup analysis, indicating renal phosphate loss *via* proximal renal tubular dysfunction.

**Conclusion:**

The AKI incidence was very low in non-ICU patients as compared to ICU patients. Hypophosphatemia is an independent risk factor for AKI in patients hospitalized for COVID-19 infection.

## Background

Proteinuria, hematuria, and acute kidney injury (AKI) have been described as common clinical presentations of renal involvement in coronavirus disease 2019 (COVID-19) patients. More than 40% of COVID-19 patients have been reported to have proteinuria and hematuria [1,2]. The incidence of AKI has ranged from 5.1% to 46% [2–6], possibly due to differences in the definition of AKI, race, rates of comorbidities, respiratory severity, and hospitalization practices.

AKI is a common complication among patients hospitalized for a wide range of diagnoses. In patients hospitalized with COVID-19, patients who developed AKI had higher mortality than those without AKI [1,7]. However, the reasons, mechanisms, and risk factors for AKI in COVID-19 patients have not been well investigated.

Multiple mechanisms for AKI in COVID-19 patients are possible, including direct virus injury, cytokine storm syndrome, antiviral and vasoconstrictor medications, hypotension, and ventilator usage. A recent study from the United States reported that mechanical ventilation was associated with AKI [7]. The onset time of AKI was related to that of mechanical ventilation suggesting that AKI may be a manifestation of disease severity of hospitalized COVID-19 patients [7,8]. The severe acute respiratory syndrome coronavirus 2 (SARS-CoV-2) virus has also been reported in autopsy kidney tissue [9]. The angiotensin-converting enzyme 2 (ACE2) and transmembrane protease serine 2 (TMPRSS2), the entry receptor for SARS-CoV-2, are highly expressed in renal tubular epithelial cells, vascular endothelial cells, and podocytes [10,11], and a spectrum of pathologic abnormalities have been identified in autopsy reports [12–15]. Although the results of renal autopsy reports cannot confirm direct infection of the virus, finding the risk factors of AKI may help explain the etiology of AKI and why patients with AKI have higher mortality.

To investigate risk factors for AKI in COVID-19 patients, we enrolled a large cohort of confirmed COVID-19 patients from four hospitals in Wuhan, China. We sought to report on AKI incidence in our cohort and identify potential risk factors for AKI in an effort to reduce AKI incidence.

## Methods

### Study cohort and design

In this retrospective study, we screened 1797 polymerase chain reaction (PCR)-confirmed COVID-19 patients who were treated in Tongji hospital (Guanggu Region), Leishenshan hospital, Renmin Hospital of Wuhan University, and Wuhan Ninth Hospital, Wuhan, Hubei, China, between February 2020 to April 2020. All patients were diagnosed as COVID-19 infected according to the standards provided by the Chinese National Health Commission (7^th^ Edition). Patients were excluded if they had less than two serum creatinine tests during their hospitalization, or if they were on maintenance dialysis or had prior kidney transplantation, or if we were unable to obtain the essential data (including missing serum creatinine, admission date, or outcome data) (Supplementary Figure 1).

The study was approved by the Institutional Review Board of the Ruijin Hospital, Shanghai Jiao Tong University School of Medicine [ETHICS No: (2020) Linlun no.34th]. The data in our cohort have not been reported for the same purpose previously.

### Data collection

Demographic characteristics, medical history, and laboratory findings at the time of admission including age, gender, comorbidities (diabetic mellitus, hypertension, cardiovascular disease [CVD], chronic obstructive pulmonary disease [COPD]), and various blood test results (neutrophil count, lymphocyte count, platelet count, alanine aminotransferase (ALT), aminotransferase (AST), albumin, urea, creatinine, uric acid, potassium, calcium, phosphorus, bicarbonate, activated partial thromboplastin time (APTT), prothrombin time (PT), D-dimer, C-reactive protein (CRP), procalcitonin (PCT), lactate dehydrogenase (LDH)) were collected. The count of missing values for laboratory findings were shown in Supplementary Table 1. Hypophosphatemia was defined as serum phosphorus less than 0.8 mmol/L. To determine AKI, all the evaluations of renal function on admission and during hospitalization were recorded. Treatments including medications (such as antiviral therapy, antibiotic therapy, or corticosteroids) and oxygen therapy (such as invasive or noninvasive mechanical ventilation) during hospitalization were noted. Patients’ outcomes (including date of transferring to intensive care unit (ICU), AKI, discharge, death, or transferring to other hospitals) were also recorded.

Tubular function evaluation including urine electrolyte, urine creatinine, urine uric acid, and protein (albumin and β2-microglobulin) excretion were measured in fifty-five patients by collecting 24-h urine samples.

### Definition of AKI

AKI was defined using KDIGO criteria [16]: an increase in serum creatinine by ≥26.5 μmol/L within 48 h or an increase in serum creatinine to ≥1.5 times baseline. Baseline serum creatinine was defined as the serum creatinine value on admission. The data of AKI onset was defined as the earliest day of a serum creatinine change meeting KDIGO criteria.

The staging of AKI was defined as follows: stage 1, increase in serum creatinine by 26.5 μmol/L within 48 h or a 1.5–1.9 times increase in serum creatinine from baseline; stage 2, 2–2.9 times increase in serum creatinine; stage 3, 3 times or more increase in serum creatinine or the initiation of renal replacement therapy [17].

### Identification of risk factors for AKI

Candidate risk factors of AKI in COVID-19 patients were selected based on clinical data and laboratory tests, including demographic characteristics (age and gender) and laboratory findings at the time of admission.

### Assessment for mechanism of hypophosphatemia

To further explore the underlying etiology for hypophosphatemia, we analyzed correlations between serum phosphorous and serum creatinine, albumin, renal phosphate loss in a subset of 55 patients.

Phosphate tubular maximum per volume of filtrate (TmP/GFR), calculated by serum phosphorus and tubular reabsorption of phosphate (TRP), is considered the best method to evaluate tubular reabsorption of phosphate and serves as an index for renal phosphate loss [17,18]. TRP was calculated using the following formula: TRP = (1-urine phosphate/urine creatinine) × (serum creatinine/serum phosphorus). TmP/GFR is dependent on the value of TRP and can be calculated using the formulas below [17]: if TRP ≤ 0.86, then TmP/GFR = TRP × serum phosphorus; if TRP > 0.86, then TmP/GFR = 0.3 × TRP/(1–0.8 × TRP) × serum phosphorus.

### Proximal renal tubular function assessment

We calculated TmP/GFR, fractional excretion of uric acid (Feur), and β2-microglobulinuria (urine β2-microglobulin-creatinine ratio [UBCR]) for each patient with hypophosphatemia. Fractional excretion of uric acid was calculated as [(urine uric acid × serum creatinine)/(urine creatinine × serum uric acid) ×100].

### Statistical analyses

Continuous variables were expressed as mean and standard deviation (SD) median, and interquartile range (IQR) values and categorical variables were described as frequencies and percentages. To compare continuous variables, independent group *t-*tests (for normally distributed data) and the Mann–Whitney test (for data that were not normally distributed) were used. Proportions for categorical variables were compared using the Chi-square or Fisher exact test. Risk factors associated with AKI were evaluated by Cox proportional hazards analysis (time from admission to AKI occurrence). The best predictive model for AKI was built by a conditional stepwise selection algorithm (entry and elimination *p* < .1), and collinearity between variables was tested. Pearson correlation was used in the association among potential factors of serum phosphorus level. Analyses were performed using Stata version 15.1 (StataCorp, College Station, Texas, USA). All statistical tests were two-sided, and *p* < .05 was considered statistically significant.

## Results

### Demographic characteristics

In total, 823 COVID-19 patients were enrolled (50.5% male) with a mean age of 60.9 ± 14.9 years. 141 (17.1%) had a history of diabetes mellitus, 329 (40.0%) had hypertension, 78 (9.5%) had CVD, and 38 (4.6%) had COPD. Until 13 April 2020, 71 (9.0%) patients were died, 648 (78.7%) patients were discharged, and 104 (12.6%) patients transferred to other hospitals. Demographic characteristics, laboratory findings, treatments, and outcomes are shown in Table 1.

**Table 1.** Demographic characteristics, laboratory findings, treatment, and outcome of enrolled COVID-19 patients, by acute kidney injury group.

	AKI	Non-AKI	Total	*P* value^a^
	(*n* = 44)	(*n* = 779)	(*n* = 823)	
Male, No. (%)	27 (61.4)	385 (49.4)	412 (50.1)	.123
Age, mean ± SD, years	72.0 ± 11.4	60.2 ± 14.9	60.9 ± 14.9	<.001
Comorbidities, No. (%)				
Diabetes Mellitus	10 (22.7)	131 (16.8)	141 (17.1)	.311
Hypertension	26 (59.1)	303 (38.9)	329 (40.0)	.008
CVD	11 (25.0)	67 (8.6)	78 (9.5)	<.001
COPD	7 (15.9)	31 (4.0)	38 (4.6)	<.001
Laboratory tests				
Neutrophil count, ×10^9^/L	8.5 ± 5.2	4.4 ± 3.1	5.6 ± 3.4	<.001
Lymphocyte count, ×10^9^/L	0.7 ± 0.3	1.5 ± 0.9	1.4 ± 0.9	<.001
Platelet, ×10^9^/L	144 (91–215)	223 (175–279)	220 (172–277)	<.001
Alanine aminotransferase, IU/L	30 (18–46)	23 (15–39)	24 (15–40)	.048
Aspartate aminotransferase, IU/L	42 (30–73)	22 (17–31)	23 (17–33)	<.001
Albumin, g/L	31.1 (27.1–33.6)	37.1 (33.4–40.2)	36.7 (32.9–40.0)	<.001
Urea, mmol/L	7.0 (4.7–11.0)	4.8 (3.8–6.1)	4.8 (3.8–6.2)	<.001
Creatinine, μmol/L	71.5 (57.0–94.3)	64.9 (54.7–79.5)	65 (55–80)	.0565
Uric acid, μmol/L	220 (134–279)	282 (230–356)	281 (225–353)	<.001
Potassium, mmol/L	4.2 ± 0.7	4.3 ± 0.6	4.3 ± 0.6	.3094
Calcium, mmol/L	2.0 ± 0.1	2.2 ± 0.1	2.2 ± 0.1	<.001
Phosphorus, mmol/L	0.81 (0.64–1.08)	1.10 (0.95–1.22)	1.09 (0.94–1.22)	<.001
Bicarbonate, mmol/L	23.0 (20.9–26.3)	24.7 (23.0–26.6)	24.7 (22.8–26.6)	.0258
Activated partial thromboplastin time, s	41.0 (32.0–51.7)	28.4 (25.3–33.1)	28.7 (25.4–33.8)	<.001
Prothrombin time, s	15.1 (12.7–17.7)	11.5 (11.0–12.5)	11.6 (11.1–12.8)	<.001
D-dimer, ug/ml	4.99 (1.30–18.36)	0.58 (0.29–1.26)	0.60 (0.30–1.40)	<.001
C-reactive protein, mg/L	110.6 (54.4–215.2)	2.5 (1.0–15.7)	2.6 (1.1–22.4)	<.001
Procalcitonin, ng/ml	0.46 (0.17–1.66)	0.05 (0.03–0.15)	0.06 (0.03–0.18)	<.001
Lactate dehydrogenase, U/L	477 (365–766)	199 (169–246)	204 (171–260)	<.001
Treatments, No. (%)				
Antiviral therapy	25 (56.8)	586 (75.2)	611 (74.2)	.007
Oseltamivir	6 (24.0)	101 (17.2)	107 (13.8)	.383
Arbidol Hydrochloride	17 (68.0)	434 (74.1)	451 (73.8)	.500
Antibiotic therapy	44 (100.0)	521 (66.9)	565 (68.6)	<.001
Corticosteroids	26 (59.1)	94 (12.1)	120 (14.6)	<.001
Mechanical Ventilation	35 (79.6)	46 (5.9)	81 (9.8)	<.001
Outcomes, No. (%)				
Discharge	2 (4.6)	646 (82.9)	648 (78.7)	<.001
Death	36 (81.8)	35 (4.5)	74 (9.0)	
Transferred	6 (13.6)	98 (12.6)	104 (12.6)	

CVD: cardiovascular disease; COPD: chronic obstructive pulmonary disease.

^a^AKI group vs. Non-AKI group.

### The incidence, onset time and outcome of acute kidney injury

AKI occurred in 44 (5.4%) patients during hospitalization, with 13 (29.5%) in KDIGO stage 1, 12 (27.3%) stage 2, and 19 (43.2%) stage 3. AKI was common in the ICU (38 cases, 40.9%) while very rare in non-ICU wards (6 cases, 0.8%). The median AKI onset time was 12 d after admission (IQR 7–17 d). The time of onset of AKI was closely related to the application of mechanical ventilation (Supplementary Figure 2). Moreover, patients with AKI had a higher probability for death than patients without AKI (unadjusted OR 95.7, 95% CI 39.3–251.8, *p* < .001; adjusted OR 107.8, 95% CI 41.0–283.6, *p* < .001, after adjustment for age, gender, and comorbidities).

### The risk factors of acute kidney injury

Next, we explored risk factors for AKI. By univariate analysis, age, hypertension, neutrophils, lymphocytes and platelets counts, alanine aminotransferase, aspartate aminotransferase, albumin, urea, calcium, phosphorus, bicarbonate, APTT, PT, d-dime, CRP, PCT, and LDH, were associated with AKI (Supplementary Table 2). Then, a forward stepwise Cox regression analysis was performed to build a predictive model for AKI, which included decreased platelet (Hazard ratio [HR] = 1.09, 95% CI 1.02–1.16), albumin (HR = 1.16, 95% CI 1.02–1.31), and phosphate levels(HR = 1.34, 95% CI 1.14–1.58), and higher LDH (HR = 1.02, 95% CI 1.00–1.03), PCT (HR = 1.06, 95% CI 1.02–1.09), CRP (HR = 1.07, 95% CI 1.02–1.12), urea (HR = 1.11, 95% CI 1.05–1.17), and PT levels (HR = 1.14, 95% CI 1.06–1.22) (Table 2, Supplementary Table 3). Linear regression was shown in phosphate level and AKI onset. For every 0.1 mmol/L decreases in serum phosphorus level, patients had a 1.34-fold increase in the risk of AKI (95% CI 1.14–1.58) after adjusting for platelet count, albumin, urea, LDH, procalcitonin, CRP, and PT.

**Table 2.** Risk factors of acute kidney injury.

	Hazard Ratio (95% CI)	*P* value
Platelet, −10 × 10^9^/L	1.09 (1.02–1.16)	.015
Albumin, −1g/L	1.16 (1.02–1.31)	.019
Urea, +1mmol/L	1.11 (1.05–1.17)	<.001
Phosphorus, −0.1mmol/L	1.34 (1.14–1.58)	<.001
Lactate dehydrogenase, + per 10U/L	1.02 (1.00–1.03)	.022
Procalcitonin, +10ng/ml	1.06 (1.02–1.09)	.001
C-reactive protein, +10mg/L	1.07 (1.02–1.12)	.010
Prothrombin time, +1s	1.14 (1.06–1.22)	<.001

^a^Forward-stepwise selection and significance level for addition to the model is 0.1.

### Hypophosphatemia and outcome

637 patients had serum phosphorate levels on admission. As serum phosphorus was a risk for AKI, we divided patients into hypophosphatemia (serum phosphorus < 0.8 mmol/L) and non-hypophosphatemia (serum phosphorus ≥ 0.8 mmol/L) groups. There were 9.4% (60/637) of patients with hypophosphatemia on admission. Demographic characteristics, laboratory findings, treatment measures, and outcomes of patients with and without hypophosphatemia at admission are shown in Table 3. Patients with hypophosphatemia were likely to be older and with lower lymphocyte count, lower serum albumin and uric acid levels, higher LDH, and CRP levels (Table 3).

**Table 3.** Demographic characteristics, laboratory findings, treatment measures, and outcome of enrolled COVID-19 patients, by phosphorus level.

	Phosphoru*s* < 0.8 mmol/L	Phosphoru*s* ≥ 0.8 mmol/L	*P* value
	(*n* = 60)	(*n* = 577)	
Male, No. (%)	37 (61.7)	284 (49.2)	.078
Age, mean ± SD, years	68.9 ± 12.8	59.9 ± 14.2	<.001
Comorbidities, No. (%)			
Diabetes Mellitus	6 (10.0)	103 (17.9)	.082
Hypertension	23 (38.3)	222 (38.5)	.550
CVD	15 (25.0)	42 (7.3)	<.001
COPD	4 (6.7)	19 (3.3)	.162
Laboratory tests			
Neutrophil count, ×10^9^/L	5.5 ± 3.6	4.3 ± 3.2	.004
Lymphocyte count, ×10^9^/L	1.0 ± 0.5	1.5 ± 1.0	<.001
Platelet, ×10^9^/L	175 (126–252)	225 (175–280)	<.001
Alanine aminotransferase, IU/L	26 (16–36)	24 (15–40)	.959
Aspartate aminotransferase, IU/L	29 (20–42)	21 (17–31)	<.001
Albumin, g/L	31.9 (30.1–35)	36.9 (33.8–39.6)	<.001
Urea, mmol/L	5.5 (4.3–7.1)	4.8 (3.8–6.2)	.004
Creatinine, μmol/L	69 (57–81)	64 (56–79)	.302
Uric acid, μmol/L	227 (157–290)	285 (231–361)	<.001
Potassium, mmol/L	4.1 ± 0.7	4.4 ± 0.6	.002
Calcium, mmol/L	2.0 ± 0.1	2.2 ± 0.1	<.001
Bicarbonate, mmol/L	24.5 (21.1–27.8)	24.7 (23.0–26.6)	.705
Activated partial thromboplastin time, s	29.7 (25.5–35.8)	28.0 (24.9–32.2)	.074
Prothrombin time, s	12.1 (11.6–13.8)	11.4 (11.0–12.3)	<.001
D-dimer, ug/ml	1.22 (0.60–4.59)	0.51 (0.24–1.28)	<.001
C-reactive protein, mg/L	37.6 (2.5–91.62)	2.11 (0.68–10.0)	<.001
Procalcitonin, ng/ml	0.06 (0.02–0.14)	0.05 (0.03–0.17)	<.001
Lactate dehydrogenase, U/L	254 (207–414)	198 (170–248)	<.001
Treatments, No. (%)			
Antiviral therapy	41 (68.3)	420 (72.8)	.276
Oseltamivir	5 (8.3)	65 (11.3)	.664
Arbidol Hydrochloride	33 (55.0)	339 (58.8)	.585
Antibiotic therapy	49 (81.7)	358 (62.0)	.005
Corticosteroids	13 (21.7)	70 (12.1)	.035
Intensive care unit	23 (38.3)	51 (8.8)	.003
Mechanical Ventilation	18 (30.0)	43 (7.5)	<.001

CVD: cardiovascular disease; COPD: chronic obstructive pulmonary disease.

**Figure 1.**
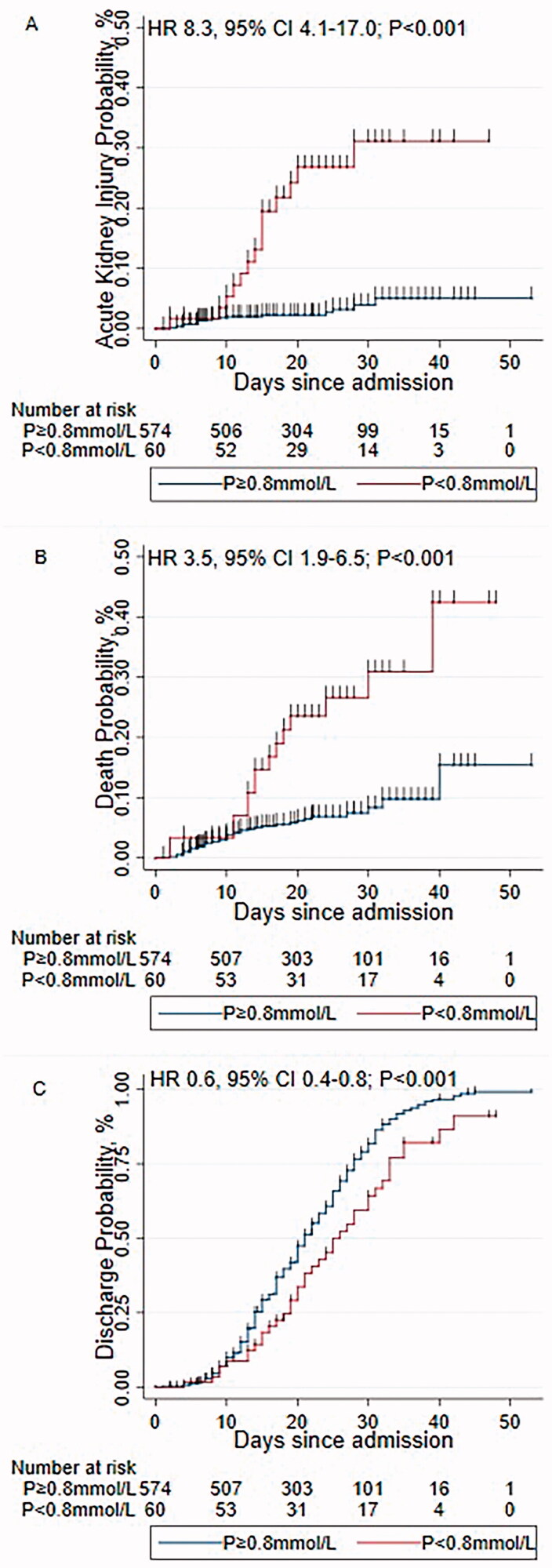
Kaplan-Meier failure curve for AKI, death, and discharge, by phosphorus level. (A) Kaplan-Meier failure curve for AKI; (B) Kaplan-Meier failure curve for death; (C) Kaplan-Meier failure curve for discharge. AKI, acute kidney injury.

Furthermore, Cox regression showed that patients with hypophosphatemia had 8.3-, 3.5- and 0.6-fold increased in risk of AKI (Figure 1(A)), death (Figure 1(B)), and discharge (Figure 1(C)), respectively.

### Evaluation for underlying cause of hypophosphatemia

We explored the reasons for hypophosphatemia in a subgroup of 55 patients for whom we were able to collect 24-h urine samples. Demographic characteristics and laboratory findings of 55 patients at admission are shown in Supplementary Table 4. Serum phosphorus level was positively correlated with TmP/GFR (Pearson *r* = 0.66, *p* < .001; Supplementary Figure 3(A)). However, no correlations were found between serum phosphorus and albumin (Pearson *r* = 0.17, *p* = .209; Supplementary Figure 3(B)) or serum creatinine levels (Pearson *r* = −0.07, *p* = .625; Supplementary Figure 3(C)).

Patients with hypophosphatemia (*n* = 5, 9.1%) had a lower TmP/GFR (*t*-test, *p* = .002; Supplementary Figure 4(A), Supplementary Table 5) and a higher Feur (*t*-test after log transformation, *p* = .002; Supplementary Figure 4(B), Supplementary Table 4) compared to patients without hypophosphatemia. No significant differences were shown in UBCR (*t-test* after log transformation, *p* = .265; Supplementary Figure 4(C), Supplementary Table 4) between patients with or without hypophosphatemia.

## Discussion

In this multicenter retrospective study, we describe an AKI incidence of 5.4% in a large COVID-19 cohort, with a much lower AKI incidence of 0.8% in non-ICU patients compared to 40.9% in ICU patients. Patients with AKI during hospitalization had a higher probability for death than those without AKI. We found that hypophosphatemia on admission was a new risk factor for AKI in hospitalized COVID-19 patients, with every 0.1 mmol/L decreases in serum phosphorus level on admission being associated with a 1.34-fold increase in the risk of AKI. We describe proximal renal tubular dysfunction as a common clinical presentation of renal dysfunction in a subset of our cohort and suggest that a potential reason for hypophosphatemia in COVID-19 patients is renal phosphate loss.

The incidence of AKI has been reported inconsistently. In a large cohort from New York City, 36.6% developed AKI during hospitalizations [7]. Here, we describe that the AKI rate was only 5.4% in COVID-19 patients, similar to the previous studies in China (5.1%) [2], but much lower than in the United States [3,7]. These differences may be due to different hospitalization practices, as we also noticed that differences in the proportion of critically ill patients among studies. The proportions of patients in the ICU or who received mechanical ventilation reported in a large cohort from New York were much higher (25.6% ICU and 21.8% received mechanical ventilation) than in our cohort, which correlated with a higher AKI rate [7]. In addition, we observed that the rate of AKI was only 0.8% in non-ICU patients, which is even lower than previous reports of 1.3% in mild or moderate severity patients [19]. Our results suggest that the rate of AKI is very low in non-critical patients with COVID-19 infection. However, the incidence of AKI is likely underestimated in our study because we could only presume that creatinine level on admission was the baseline for patients. Consistent with previous studies, we observed a poorer prognosis in COVID-19 patients with AKI.

Moreover, we report that decreased platelet and albumin levels, increased urea, prolonged PT, elevated d-dime, and inflammatory factors (CRP, PCT, and LDH) were associated with AKI. These variables are known risk factors for disease severity [20] and in-hospital mortality [4,20–24]. Our results support that AKI development is correlated to disease severity, which may explain the higher rate of AKI in ICU patients.

We found that 10% of patients had hypophosphatemia on admission and that hypophosphatemia was an independent risk factor for subsequent development of AKI in hospitalized COVID-19 patients. Hypophosphatemia, considered as a general marker of illness severity in critically ill patients [25], was found in about 20–50% of ICU patients on admission. Previous studies have reported that hypophosphatemia may impair the contractile properties of the diaphragm during acute respiratory failure [26], although correction of hypophosphatemia has not been shown to decrease mortality [25,27–29]. Our results show that patients with hypophosphatemia had lower lymphocyte count, higher aspartate aminotransferase, longer prothrombin time, higher CRP and LDH levels, which suggests that hypophosphatemia may have been related to disease severity and multiple organ injuries. Some studies reported that admission serum phosphate over 4.4 mg/dl and 4.9 mg/ml were associated with an increased risk of developing AKI [30] and in-hospital mortality [31] in hospitalized patients. However, we did not observe this correlation in patients with COVID-19 in our study. It may due to limited cases with hyperphosphatemia in our study. Phosphorus metabolism is complex in critically ill patients and can be affected by renal function, nutritional status [32], and renal phosphorus loss [29,33]. In a subset of our cohort, we found that 27.0% of patients had proximal tubule dysfunction, supporting renal tubular injury was common in COVID-19 patients.

To date, the mechanism for AKI in COVID-19 has not been well elucidated. Several histologic findings were observed in COVID-19 patients, including acute tubular injury, collapsing glomerulopathy, and endothelial injury/thrombotic microangiopathy [34]. Previous studies suggest that SARS-CoV-2 may cause cell damage through direct infection of the kidney. As the ACE2 receptor for SARS-CoV-2 is highly expressed in the kidney [35], direct viral tissue damage is a plausible mechanism of injury. In addition, endothelial damage and thromboinflammation, dysregulation of immune responses, and maladaptation of ACE2-related pathways might all contribute to these extrapulmonary manifestations of COVID-19 [36]. SARS-CoV-2 RNA has also been detected in the urine [37], and viral particles have also been identified in the epithelium of proximal tubules by electronic microscopy [15,38]. However, identification of viral particles is difficult and can be confounded by similar appearances of organelles as reported by Goldsmith CS [39], and not all reported data are consistent. In one recent case series of hospitalized COVID-19 with AKI, acute tubular injury and epithelial necrosis in the biopsied kidney tissue were the predominant histopathologic findings which are similar to that of septic AKI [13,14]. Glomerular disease presenting as proteinuria with or without AKI was also considered as an important presentation of COVID-19 infection in patients with a high-risk APOL1 genotype [40].

Proximal tubule dysfunction has also been reported in hospitalized COVID-19 patients: one prior study found that approximately 50% of patients had inappropriate uricosuria or phosphaturia [41]. In our study, decreased phosphate reabsorption rate was associated with hypophosphatemia, suggesting renal phosphate loss in patients with COVID-19. Furthermore, the proximal renal tubular injury was more severe in patients with hypophosphatemia. Although our results cannot distinguish whether the renal tubular injury is caused by a direct viral infection, hypoperfusion, medication, or inflammatory factors after viral infection, our study suggests renal phosphate loss may be an important contributor to hypophosphatemia.

Nutritional status is also a cause of hypophosphatemia. We found that a lower albumin level was presented in patients with hypophosphatemia. Symptoms such as anorexia, nausea, and vomiting, presented in many COVID-19 patients, can lead to inadequate intake. Internal redistribution of phosphorous, as a result of increased insulin secretion, may also contribute [42]. Our findings supported that hypophosphatemia was associated with the general condition and disease severity for COVID-19 infection.

Our study has several strengths. First, our study enrolled a large cohort of 823 hospitalized patients with COVID-19 with strong data integrity, and we were able to evaluate relatively comprehensive demographic and clinical contributors for AKI in our cohort. Second, we were able to conduct a comprehensive assessment of renal tubular function in subgroup analysis, including TmP/GFR, fractional excretion of uric acid, and urine β2-microglobulin-creatinine ratio. Last, our study confirmed proximal tubule dysfunction as a kind of clinic presentation for kidney injury in COVID-19 patients and potentially suggests virus direct infection of the kidney.

We acknowledge several limitations of our study. The incidence of AKI may be underestimated in our study because we did not have data about pre-admission baseline renal function and could only presume that creatinine level on admission was the baseline. We only captured hospitalized-acquired AKI in our study. However, the risk of worsening renal function (or new AKI) during hospitalization is still important. In addition, evaluation of renal tubular function was only available in a small group of patients due to our retrospective study design. Therefore, the findings of this study should be validated. Last, due to the retrospective design, patients’ history of chronic kidney disease, gastrointestinal diseases, or other chronic diseases might be incomplete. Besides, our study cannot establish the causality between hypophosphatemia and AKI onset.

In a conclusion, AKI that developed after hospitalization was common in critically ill patients but rare in non-critical patients with COVID-19. We identified hypophosphatemia as an independent risk factor for AKI among COVID-19 patients, and our data suggest that renal tubular dysfunction may contribute to the underlying etiology.

## Supplementary Material

Supplemental Material

Supplemental Material

## Data Availability

The datasets used and/or analyzed during the current study are available from the corresponding author on reasonable request.

## Abbreviations

AKI: Acute kidney injury
COVID-19: Coronavirus disease 2019
ACE2: Angiotensin-converting enzyme 2
ALT: Alanine aminotransferase
APTT: Activated partial thromboplastin time
AST: Aminotransferase
COPD: Chronic obstructive pulmonary disease
CRP: C-reactive protein
CVD: Cardiovascular disease
Feur: Fractional excretion of uric acid
HR: Hazard ratio
ICU: Intensive care unit
IQR: Interquartile range
LDH: Lactate dehydrogenase
PCR: Polymerase chain reaction
PCT: Procalcitonin
PT: Prothrombin time
SARS-CoV-2: Severe acute respiratory syndrome coronavirus 2
TmP/GFR: Phosphate tubular maximum per volume of filtrate
TMPRSS2: Transmembrane protease serine 2
TRP: Tubular reabsorption of phosphate
UBCR: Urine β2-microglobulin-creatinine ratio
