# Supplementary material for: Hypophosphatemia is an independent risk factor for AKI among hospitalized patients with COVID-19 infection
Source: Ren Fail. 2021 Sep 19;43(1):1329–37. doi: 10.1080/0886022X.2021.1979039 (PMC8462927; doi:10.1080/0886022X.2021.1979039)
Supplement: Supplemental Material [file IRNF_A_1979039_SM5809.pdf]

Supplementary Table 1 The count of missing value for laboratory findings among 823 COVID-19 patients

Laboratory tests	Count of missing value
**Neutrophil count, ×10^9/L**	3
**Lymphocyte count, ×10^9/L**	3
**Platelet, ×10^9/L**	4
**Alanine aminotransferase, IU/L**	2
**Aspartate aminotransferase, IU/L**	2
**Albumin, g/L**	2
**Urea, mmol/L**	0
**Creatinine, µmol/L**	0
**Uric acid, µmol/L**	0
**Potassium, mmol/L**	1
**Calcium, mmol/L**	3
**Phosphorus, mmol/L**	186
**Bicarbonate, mmol/L**	153
**Activated partial thromboplastin time, s**	77
**Prothrombin time, s**	77
**D-dimer, ug/ml**	87
**C-reactive protein, mg/L**	96
**Procalcitonin, ng/ml**	112
**Lactate dehydrogenase, U/L**	21

Supplementary Table 2 Risk factors for acute kidney injury in COVID-19 patients

	**Hazard Ratio (95% CI)**	***P* value**	**Harrell's C**
Age, +1 yr	1.07 (1.04-1.09)	<0.001	0.708
Male, vs female	1.6 (0.87-2.93)	0.131	0.553
Diabetes, yes vs no	1.31 (0.65-2.66)	0.449	0.517
Hypertension, yes vs no	2.17 (1.19-3.96)	0.011	0.603
Neutrophil count, $+1\times10^9$/L	1.16 (1.12-1.20)	<0.001	0.781
Lymphocyte count , -1×10^9/L	15.43 (7.33-32.47)	<0.001	0.851
Platelet, $-10\times\times10^9$/L	1.10 (1.06-1.15)	<0.001	0.744
Alanine aminotransferase, +100U/L	1.08 (1.03-1.13)	0.001	0.593
Aspartate aminotransferase, +100U/L	1.05 (1.02-1.08)	<0.001	0.808
Albumin, -1g/L	1.24 (1.18-1.31)	<0.001	0.833
Urea, +1mmol/L	1.05 (1.03-1.07)	<0.001	0.727
Creatinine, -10umol/L	1.01 (0.98-1.03)	0.634	0.390
Uric acid, -10umol/L	1.03 (0.99-1.06)	0.066	0.644
Calcium, -0.1mmol/L	1.82 (1.52-2.18)	<0.001	0.781
Phosphorus, -0.1mmol/L	1.41 (1.181.67)	<0.001	0.695
Bicarbonate, +1mmol/L	0.89 (0.83-0.95)	<0.001	0.601
Lactate dehydrogenase, + per 10U/L	1.03 (1.02-1.03)	<0.001	0.919
Procalcitonin, +10ng/ml	1.04 (1.01-1.07)	0.003	0.869
C-reactive protein, +10mg/L	1.15 (1.12-1.18)	<0.001	0.903
Activated partial thromboplastin time, +1s	1.04 (1.02-1.04)	<0.001	0.762
Prothrombin time, +1s	1.11 (1.09-1.14)	<0.001	0.838
D-dimer, +1ug/ml	1.05 (1.03-1.07)	<0.001	0.826

Supplementary Table 3. The variance inflation factor for variables associated with acute kidney injury

Variable	Variance inflation factor
Platelet	1.91
Albumin	1.55
Urea	1.54
Phosphorus	1.50
Lactate dehydrogenase	1.41
Procalcitonin	1.30
C-reactive protein	1.08
Prothrombin time	1.01

Supplementary Table 4. Demographic characteristics and laboratory findings of 55 patients on admission

	Hospitalized COVID-19 patients (n=55)
Male, No. (%)	24(43.6)
Age, mean±SD, years	61.5±12.5
**Comorbidities, No.(%)**	
Diabetes Mellitus	16(29.1)
Hypertension	26(47.3)
CVD	4(7.3)
COPD	4(7.3)
**Laboratory tests**	
Neutrophil count, $\times 10^9$/L	5.3±3.6
Lymphocyte count, $\times 10^9$/L	1.2±0.6
Platelet, $\times 10^9$/L	229(180-285)
Alanine aminotransferase, IU/L	24(14-45)
Aspartate aminotransferase, IU/L	34(30-39)
Albumin, g/L	34.0(30.0-39.0)
Urea, mmol/L	4.2(3.6-5.7)
Creatinine, μmol/L	67(55-81)
Uric acid, μmol/L	231(164-281)
Potassium, mmol/L	4.2±0.6
Calcium, mmol/L	2.1±0.1
Phosphorus, mmol/L	1.10(0.94-1.17)
Bicarbonate, mmol/L	23.7(21.7-25.6)
Activated partial thromboplastin time, s	40(37.2-45.9)
Prothrombin time, s	13.7(13.4-14.7)
D-dimer, ug/ml	0.76(0.39-2.95)
C-reactive protein, mg/L	21.1(3.4-101.5)
Procalcitonin, ng/ml	0.06(0.06-0.11)
Lactate dehydrogenase, U/L	245(196-400)

Supplementary Table 5. The proximal tubular function of 55 hospitalized COVID-19 patients, by serum phosphorus level

	Total	Phosphorus <0.8mmol/L	Phosphorus ≥ 0.8mmol/L
**N**	55	5	50
**TmP/GFR, mmol/L**	0.94±0.29	0.57±0.11	0.98±0.28
**N**	55	5	50
**Feur, %**	9.71(7.54,13.18)	21.01(16.84,26.93)	9.40(7.50,12.24)
**N**	21	4	17
**UBCR, ug/g**	1890.2(356.4,45865.9)	25810.7(3264.0,95945.5)	877.6(224.3,40401.7)

TmP/GFR, tubular maximum for phosphate corrected for the glomerular filtration rate; Feur, fractional excretion of uric acid; UBCR, urine β2-microglobulin-creatinine ratio.
