# Supplementary material for: Hypophosphatemia is an independent risk factor for AKI among hospitalized patients with COVID-19 infection
Source: Ren Fail. 2021 Sep 19;43(1):1329–37. doi: 10.1080/0886022X.2021.1979039 (PMC8462927; doi:10.1080/0886022X.2021.1979039)
Supplement: Supplemental Material [file IRNF_A_1979039_SM5766.pdf]

Supplementary Figure 1. The flow chart inclusion criteria of patients in our study

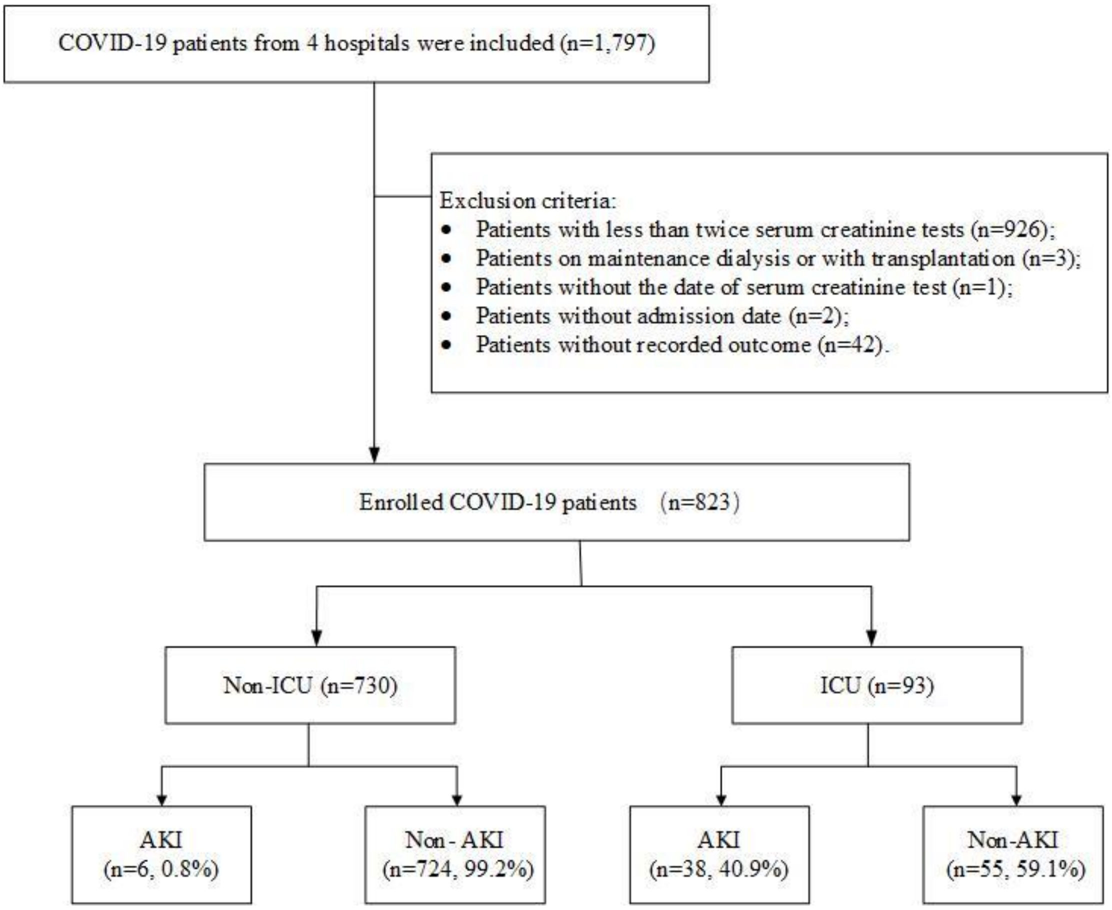

Supplementary Figure 2. The cumulative number of patients with AKI and mechanical ventilation in COVID-19 patients, by hospital day of admission

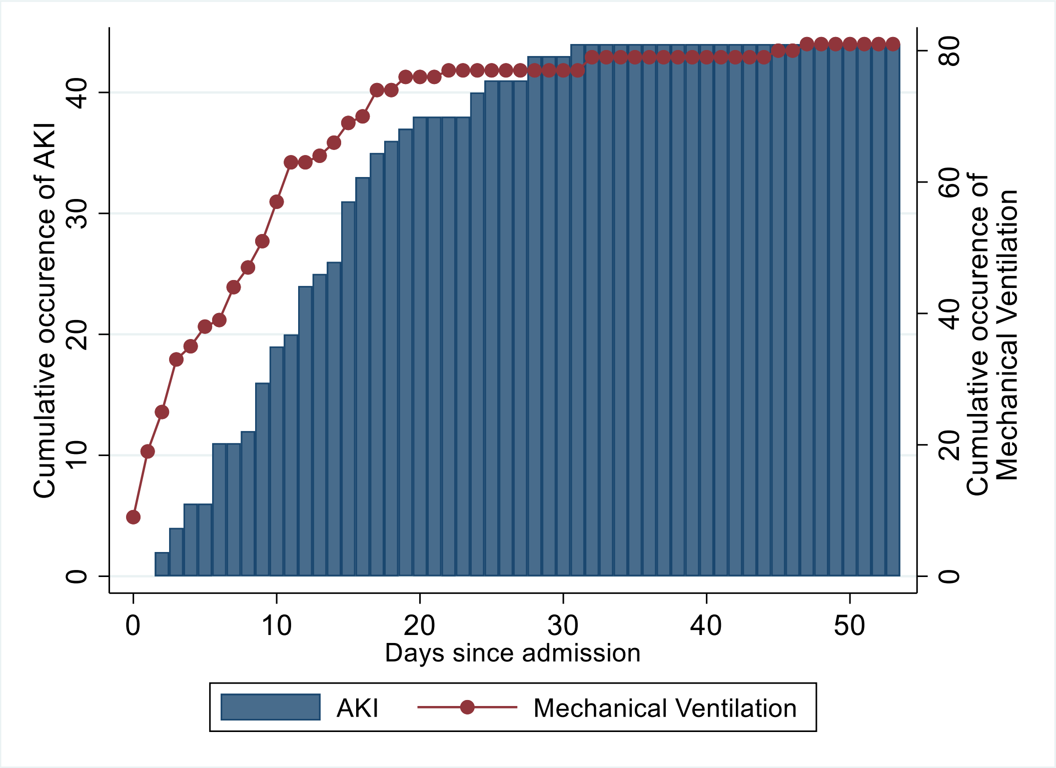

Supplementary Figure 3. Scatterplots of serum phosphorus level, TmP/GFR, albumin, and creatinine

A) Scatterplot of serum phosphorus level and TmP/GFR;

B) Scatterplot of serum phosphorus level and albumin;

C) Scatterplot of serum phosphorus level and creatinine.

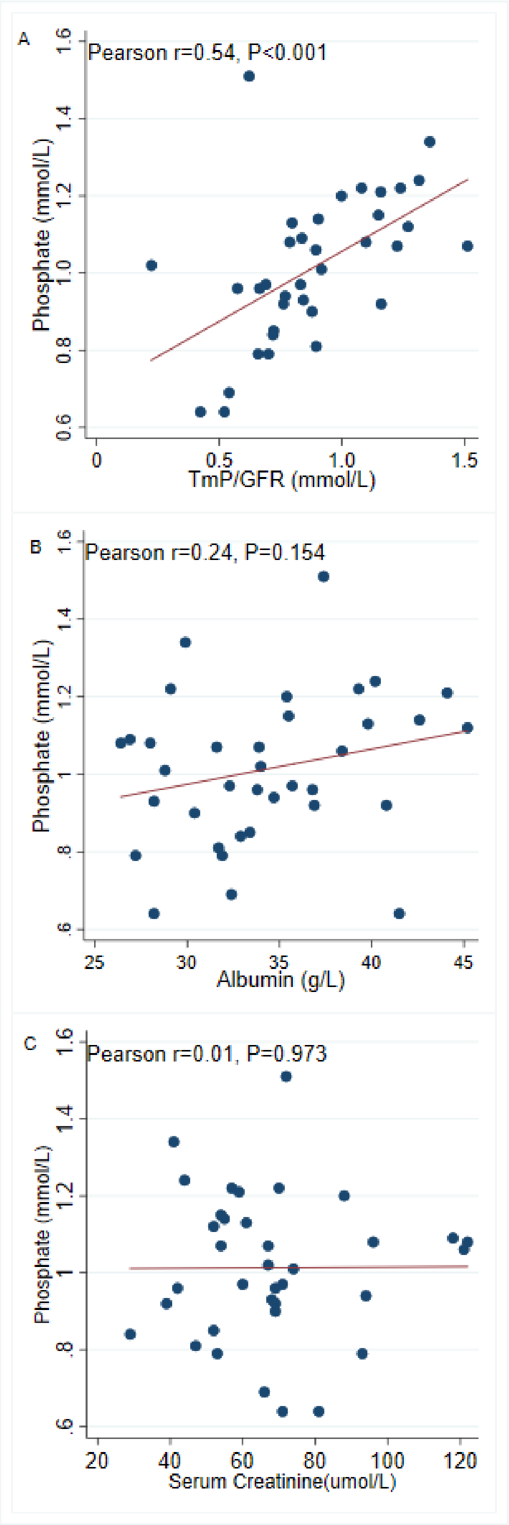

TmP/GFR, phosphate tubular maximum per volume of filtrate.

Legend: In 55 COVID-19 patients, serum phosphorus level was positively correlated with TmP/GFR (Pearson r=0.66, P<0.001). No correlations were found between serum phosphorus and albumin or serum creatinine level.

Supplementary Figure 4. Dot plots of the proximal tubular function of 55 hospitalized COVID-19 patients, by serum phosphorus level

A) Dot plot of TmP/GFR by serum phosphorus level;

B) Dot plot of Feur by serum phosphorus level;

C) Dot plot of UBCR by serum phosphorus level.

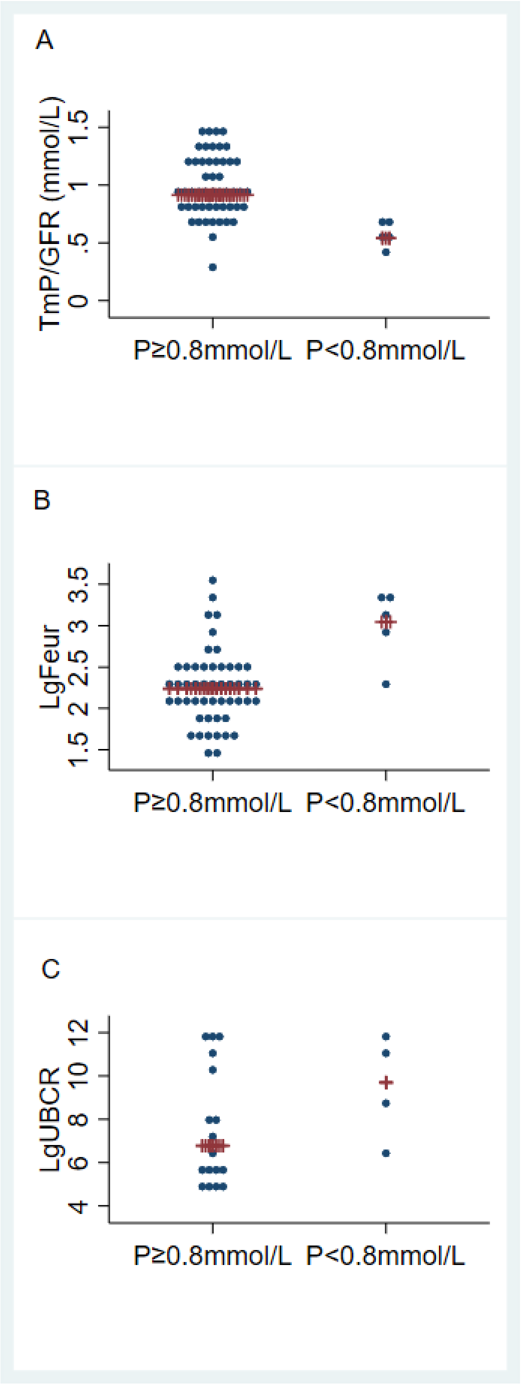

TmP/GFR, phosphate tubular maximum per volume of filtrate; Feur, fractional excretion of uric acid; UBCR, urine β2-microglobulin-creatinine ratio.

Legend: The values and the mean of the proximal tubular function for each patient were shown in figures. We compared TmP/GFR, Feur, and UBCR by serum phosphorus level. As Feur and UBCR were not normal distribution, *t-test* was performed after log transformation. TmP/GFR and LgFeur was significant lower in hypophosphatemia patients, while no significant difference was shown in LgUBCR.
